# Morphological effect of oscillating magnetic nanoparticles in killing tumor cells

**DOI:** 10.1186/1556-276X-9-195

**Published:** 2014-04-28

**Authors:** Dengfeng Cheng, Xiao Li, Guoxin Zhang, Hongcheng Shi

**Affiliations:** 1 Department of Nuclear Medicine, Zhongshan Hospital, Fudan University, Shanghai 200032, People's Republic of China; 2 Shanghai Institute of Medical Imaging, Shanghai 200032, People's Republic of China; 3 Shanghai Institute of Applied Physics, Chinese Academy of Sciences, Shanghai 201800, People's Republic of China

**Keywords:** Mechanical damage, Morphological effect, Forced oscillation, Magnetic nanoparticles, Cell viability, Alternating magnetic field

## Abstract

Forced oscillation of spherical and rod-shaped iron oxide magnetic nanoparticles (MNPs) via low-power and low-frequency alternating magnetic field (AMF) was firstly used to kill cancer cells *in vitro*. After being loaded by human cervical cancer cells line (HeLa) and then exposed to a 35-kHz AMF, MNPs mechanically damaged cell membranes and cytoplasm, decreasing the cell viability. It was found that the concentration and morphology of the MNPs significantly influenced the cell-killing efficiency of oscillating MNPs. In this preliminary study, when HeLa cells were pre-incubated with 100 μg/mL rod-shaped MNPs (rMNP, length of 200 ± 50 nm and diameter of 50 to 120 nm) for 20 h, MTT assay proved that the cell viability decreased by 30.9% after being exposed to AMF for 2 h, while the cell viability decreased by 11.7% if spherical MNPs (sMNP, diameter of 200 ± 50 nm) were used for investigation. Furthermore, the morphological effect of MNPs on cell viability was confirmed by trypan blue assay: 39.5% rMNP-loaded cells and 15.1% sMNP-loaded cells were stained after being exposed to AMF for 2 h. It was also interesting to find that killing tumor cells at either higher (500 μg/mL) or lower (20 μg/mL) concentration of MNPs was less efficient than that achieved at 100 μg/mL concentration. In conclusion, the relatively asymmetric morphological rod-shaped MNPs can kill cancer cells more effectively than spherical MNPs when being exposed to AMF by virtue of their mechanical oscillations.

## Background

Due to their excellent biocompatibility, monodispersity, and magnetic resonance, iron oxide (Fe_3_O_4_) magnetic nanoparticles (MNPs) have been proved useful in various biomedical applications such as contrast agent in magnetic resonance imaging [1], cellular imaging [2], drug carrier in targeted drug delivery system [3,4], and magnetic fluids in hyperthermia [5,6].

Alternating magnetic field (AMF)-assisted thermal therapy has received widespread attention for tumor treatment recently. In thermal therapy, the localized MNPs in cancerous tissue generate abundant heat via a high-frequency (300 to 1,100 kHz) AMF for an irreversible thermal injury [7,8]. However, the neighboring healthy tissues may also be injured by the redundant heat. It is proved that the heat generation efficiency of MNPs heavily depends on the particle size and frequency of external AMF [7,9]. As the particle size increases to micron-sized or AMF frequency decreases, the degree of Néel relaxation and Brownian relaxation decreases, suppressing heat generation. Meantime, AMF-induced vibration or rotation of particles displaces heat generation as the main pattern of AMF energy consumption. In a newly reported research, magnetic microdiscs were used for targeted cancer cell destruction by means of AMF-induced vibrations [10]. In theory, the MNPs reorient in the alternating magnetic field [11] and the oscillation of immobilized MNPs takes place *in situ* in the localization of cancerous tissues [12]. Hence, the oscillating MNPs can mechanically damage cancerous tissues at the cellular level as ‘nanoscale scalpel’. It is notable that no thermal damage will be made to the surrounding tissues.

The utilization of forced vibration of MNPs makes the best use of the neglected part of AMF energy consumption. In biomedical applications of forced MNP vibration, patterns and intensity of MNPs' vibration, as well as the degree of thermal damage, will vary according to differences in size, morphology, and exposure concentration of MNPs. By now, most biomedical application research of MNPs related to nanospheres [13]. However, the involvement of rod-shaped MNPs (rMNP) is greater than that of spherical MNPs (sMNP). In this research, an assumption that AMF-induced oscillations of rMNPs can damage cell viability more seriously will be investigated *in vitro* on human cervical carcinoma cells (HeLa), considering their extensive use in cells uptake and tumor therapy research [14-16]. Similarly sized rod-shaped (length 200 ± 50 nm, diameter 50 to 120 nm) and spherical (diameter 200 ± 50 nm) Fe_3_O_4_ MNPs in three different concentrations were synthesized and used to investigate the effects of MNP morphology and concentration in killing tumor cells.

## Methods

### Synthesis of MNPs

*Spherical Fe*_*3*_*O*_*4*_*MNPs* FeCl_3_ · 6H_2_O (0.81 g) was dissolved in 25 mL glycol and transferred to a 50-mL teflon-lined stainless steel autoclave. KAc (1.47 g) was then added to the solution, stirring constantly. Autoclave was sealed and maintained at 200°C for 24 h. After naturally cooled to room temperature, the black magnetite particles were gathered by magnet and washed with deionized water and ethanol three times, respectively. The final product was dried in a vacuum at 60°C for 12 h.

*Rod-shaped Fe*_*3*_*O*_*4*_*MNPs* Rod-shaped MNPs were synthesized following the procedure described previously [17]. Stoichiometric FeSO_4_ · 7H_2_O (0.139 g), FeCl_3_ · 6H_2_O (0.270 g), and 5 mL ethylenediamine were sealed in the autoclave and maintained at 120°C for 12 h. After naturally cooled to room temperature, the rMNPs were gathered by magnet and then post-processed with the same steps for sMNPs.

The prepared MNPs were ultrasonically treated to break up clusters and then sterilized using 75% (*v*/*v*) ethanol. The sterile MNPs were dissolved in DMEM medium at concentrations of 20, 100, and 500 μg/mL.

### Material characterization: TEM, XRD, and VSM

Morphology and size of MNPs were observed by transmission electron microscopy (TEM) (H-800; Hitachi, Chiyoda, Tokyo, Japan) operating at 200 kV. Composition and crystal form were characterized by X-ray diffraction (XRD) (D/MAX 2200; Rigaku, Tokyo, Japan) with Cu Kα radiation (λ = 0.154056 nm), with operation voltage at 40 kV and current at 40 mA. Magnetic properties including the saturation magnetic induction and coercivity were measured by vibrating sample magnetometer (VSM) (Lakeshore 7407; Lake Shore Cryotronics Inc., Westerville, OH, USA).

### AMF-generating device

**Figure 1:**
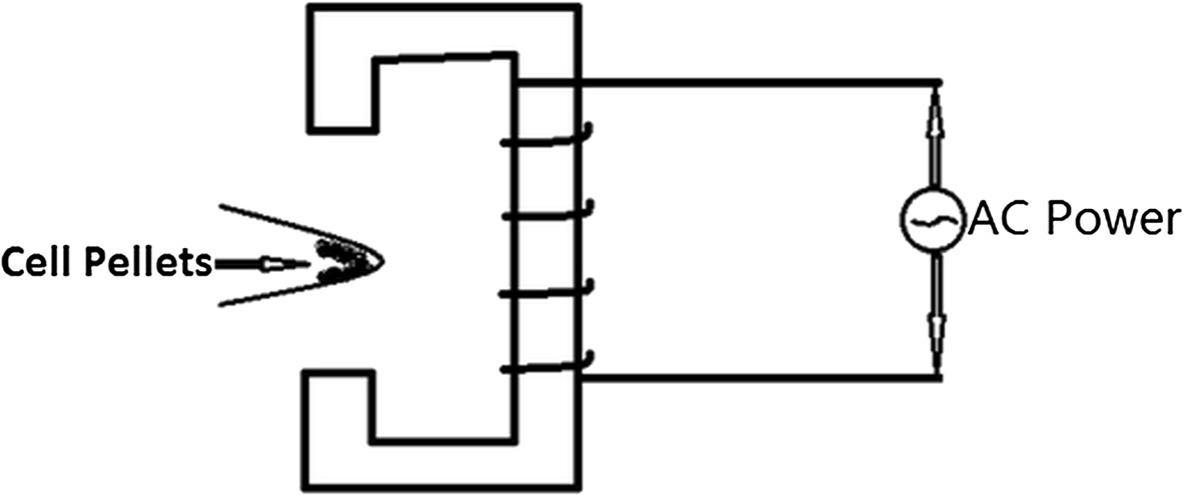
**Schematic diagram of alternating magnetic field.** Cell pellets are placed between the two ends of AMF.

The AMF-generating device was made in-house following the schematic diagram in Figure 1. A 50-Hz alternating current was transformed into a direct current and then into a 35-kHz alternating current. The alternating current acted on a U-shaped iron core to generate a stable alternating magnetic field between the two ends. The effective power (0.3 W) of this device is lower than the commonly used thermal therapy heating devices but is sufficient to make the MNPs vibrate in AMF.

### Quantification of MNPs' loading

HeLa cells (Cell Bank at the Chinese Academy of Science, Shanghai, China) were seeded at a density of 10^4^ cells/well in a 96-well plate. After 2 h incubation at 37°C in 5% CO_2_ atmosphere, the cells were exposed to the culture medium containing MNPs at concentrations of 20 (low), 100 (medium), or 500 μg/mL (high) for 3, 6, 12, or 20 h. At four desired time points, cells were rinsed with phosphate-buffered saline (PBS) to remove unfixed MNPs. Then, the MNP-loaded cells in each well were fully dissolved by hydrochloric acid (37.5%, *w*/*v*). At last, ferrozine solution (10 mg/mL) was added, and the absorbance of complex of ferrozine and ferrous ion was measured using spectrophotometer (UV 3100; Shanghai Mapada Intruments Co., Ltd., Shanghai, China). Ferrous ions were quantified by referencing the corresponding standard curve.

### Treatment of MNP-loaded HeLa cells

HeLa cells were cultured in 50 mL tissue culture flask at 37°C in 5% CO_2_ atmosphere with three concentrations of MNPs as stated above, and the optimized incubation time was selected based on the quantification results. After incubation, the cells were rinsed with PBS twice to remove the unfixed MNPs. Then, the dissociated cells were equally divided into five 0.5-mL centrifuge tubes and centrifuged at 1,000 rpm for 3 min. Finally, the cell pellet was placed in the center of AMF with culture medium covering the cells and then treated by the AMF device for 0, 10, 30, 60, or 120 min, respectively. AMF treatments of MNPs and MNP-loaded cells were performed at 37°C in airtight conditions. The temperature of cell pellet was recorded by the infrared thermometer (OS 3708; Omega Engineering, Stamford, CT, USA).

### Cell viability assay: MTT assay and trypan blue assay

*MTT assay* Cell viability was measured using 3-(4,5-dimethylthiazol-2-yl)-2,5-diphenyltetrazolium bromide (MTT; Sigma-Aldrich Company Ltd., Gillingham, Dorset, UK) assay. After being treated in AMF, HeLa cells were reseeded into 96-well petriplate for 2 h incubation in quintuplicate. Following incubation, 20 μL MTT (5 mg/mL in PBS) solution was added to each well and incubated for another 4 h. After that, the culture supernatant was extracted, and purple insoluble MTT product was re-dissolved in 150 μL dimethyl sulfoxide. Lastly, the concentration of the reduced MTT in each well was measured at 570 nm using a microplate reader. It is notable that the untreated MNP-loaded cells (i.e., the 0 min group) were used as control and absorbance was adjusted by correcting for the bias caused by the dark MNPs.

*Trypan blue assay* After being treated with AMF, the medium was removed and the cells were stained by 0.4% trypan blue (Sigma-Aldrich Company Ltd., Gillingham, Dorset, UK) solution for 3 min. The cells with damaged cell membranes were stained by trypan blue and counted under the optical microscope. The above tests were repeated three times.

### Optical images of cellular semi-thin sections, SEM of cell surface, and TEM of cellular ultramicrocuts

The HeLa cells were firstly fixed by adding 0.5% and 2% (*w*/*v*) glutaraldehyde and kept for 1 h at room temperature. Then the cells were dehydrated with ethanol in series of concentrations 50%, 70%, 80%, 90%, and 100% (*v*/*v*) for 10 min respectively. Finally, the acetone-infiltrated cells were embedded in resin, and the blocks containing the cells were cut into thin sections in 500 or 50 nm using a diamond knife. For TEM of internal cell structure, the 50-nm ultramicrocuts were transferred into a copper grid for viewing. For optical macroscope viewing (6XB-PC, Shanghai Optical Instrument Factory, Shanghai, China), the 500-nm semi-thin sections were observed. For scanning electron microscope (SEM; LEO1530VP; LEO Elektronenmikroskopie GmbH, Oberkochen, Germany) of cell surfaces, the dehydrated cells were conductively coated and observed at 5 kV.

## Results and discussion

### Materials characterization

**Figure 2:**
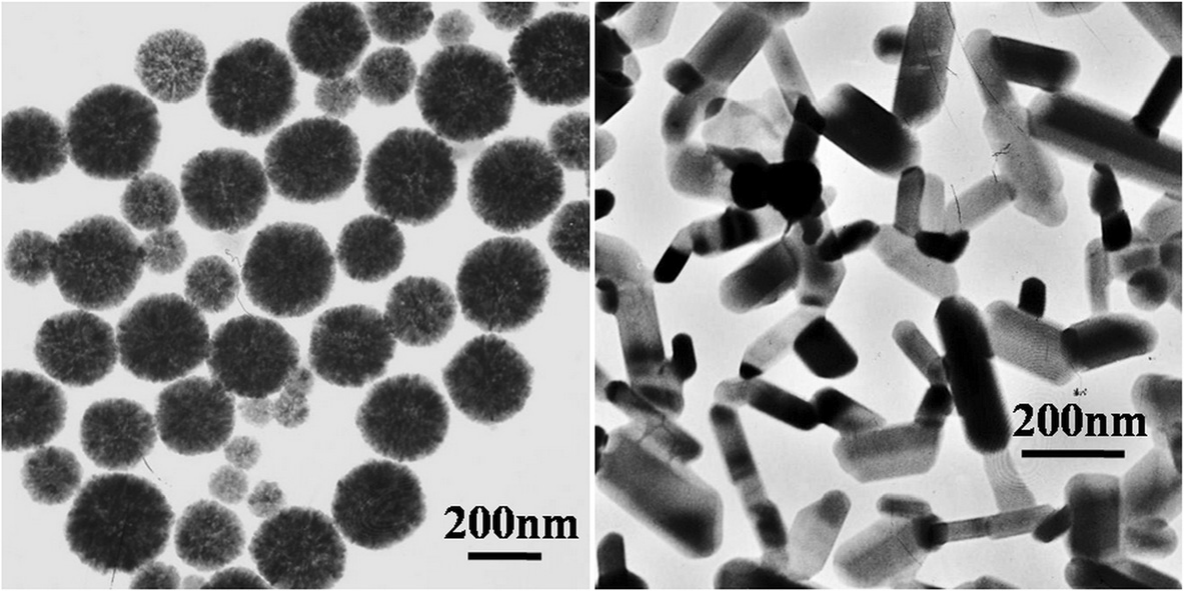
TEM images of spherical (left) and rod-shaped (right) iron oxide MNPs used.

TEM images of MNPs (Figure 2) revealed that most spherical MNPs were of a diameter of 200 ± 50 nm, while minority of MNPs was smaller. For rod-shaped MNPs, length was 200 ± 50 nm and diameters ranged from 50 to 120 nm. XRD patterns revealed that both types of MNPs were pure Fe_3_O_4_ (JCPDS no 19-0629). Meanwhile, the relatively strong (311) peak of rod-shaped MNPs implied that the crystals grow along the (311) crystallization plane to form rods. The saturation magnetic inductions for the MNPs were similar: 70.347 emu/g for sMNPs and 74.971 emu/g for rMNPs. The coercivity of the rod-shaped MNPs was 110.42 Gs, while the coercivity of the spherical MNPs was 53.18 Gs.

### Thermal effect of AMF

During the AMF treatment, neither type of MNPs leads to an obvious temperature rise. This is because of the low power and low frequency of the device relative to the commonly used thermal therapy device [18,19]. When 0.1 g solid MNPs powder was placed in the center of the AMF-generating device, the maximal temperature rise was 1.7°C. It is known that the required temperature for irreparable cell damage during hyperthermia therapy should be no less than 43°C [20,21]. Additionally, the relative small mass fraction of MNPs was used in the treated unit. Therefore, this marginal temperature rise suggested that the thermal injury could be neglected in this study.

### Cell cytotoxicity and characterization of cell loading

**Figure 3:**
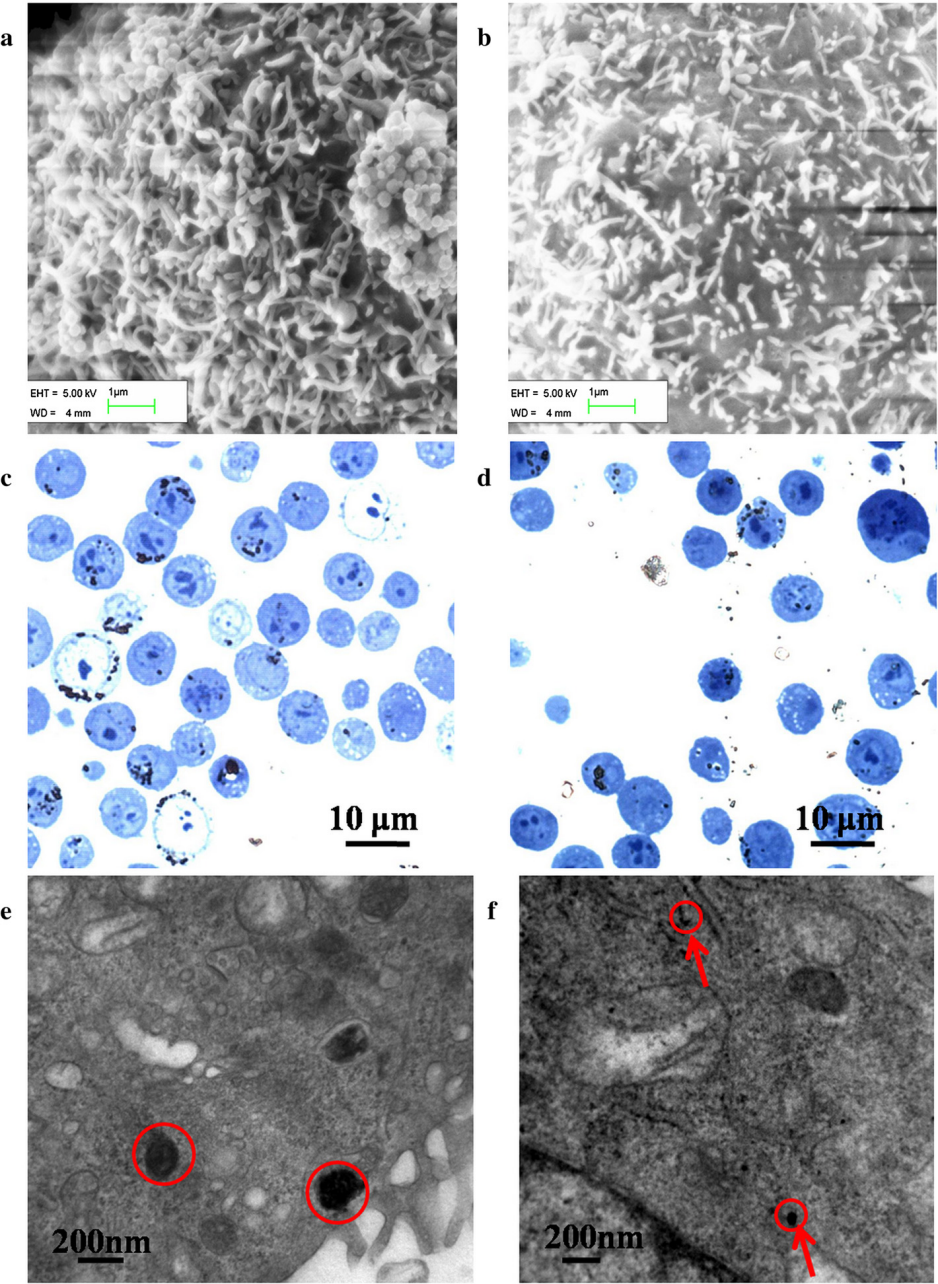
**Images of MNP-loaded HeLa cell. (a,b)** SEM images of HeLa cell membranes showing MNPs loading. **(c,d)** Optical microscopy images of semi-thin sections (500 nm thicker than the MNPs' diameter). **(e,f)** TEM images of cell ultramicrocuts (50 nm thinner than the diameter or width of MNPs); The arrows in **(f)** point to cut rod-shaped MNPs in the ultramicrocuts.

HeLa cells incubated with either spherical or rod-shaped MNPs exhibited no signs of toxicity at any of the three concentrations. Meanwhile, the rMNPs promoted cell proliferation slightly, as like as the results of previous research by Tomitaka et al*.*[22]. After 20 h incubation in medium containing MNPs, the amount of MNP intake by the single cell reached the peak. sMNPs (85%) and rMNPs (89%) were loaded by HeLa cells at the concentration of 100 μg/mL. As shown in Figure 3a,b, abundant MNPs were embedded in the HeLa cell membrane. The majority of the MNPs are distributed evenly while the minority forming clusters. Optical images (Figure 3c,d) showed that majority of MNPs are distributed on the cellular surfaces. TEM images of cell ultramicrocuts (Figure 3e,f) revealed that part of the MNPs were incorporated into the cells' cytoplasma and were distributed evenly.

### Cell viability after AMF treatment

**Figure 4:**
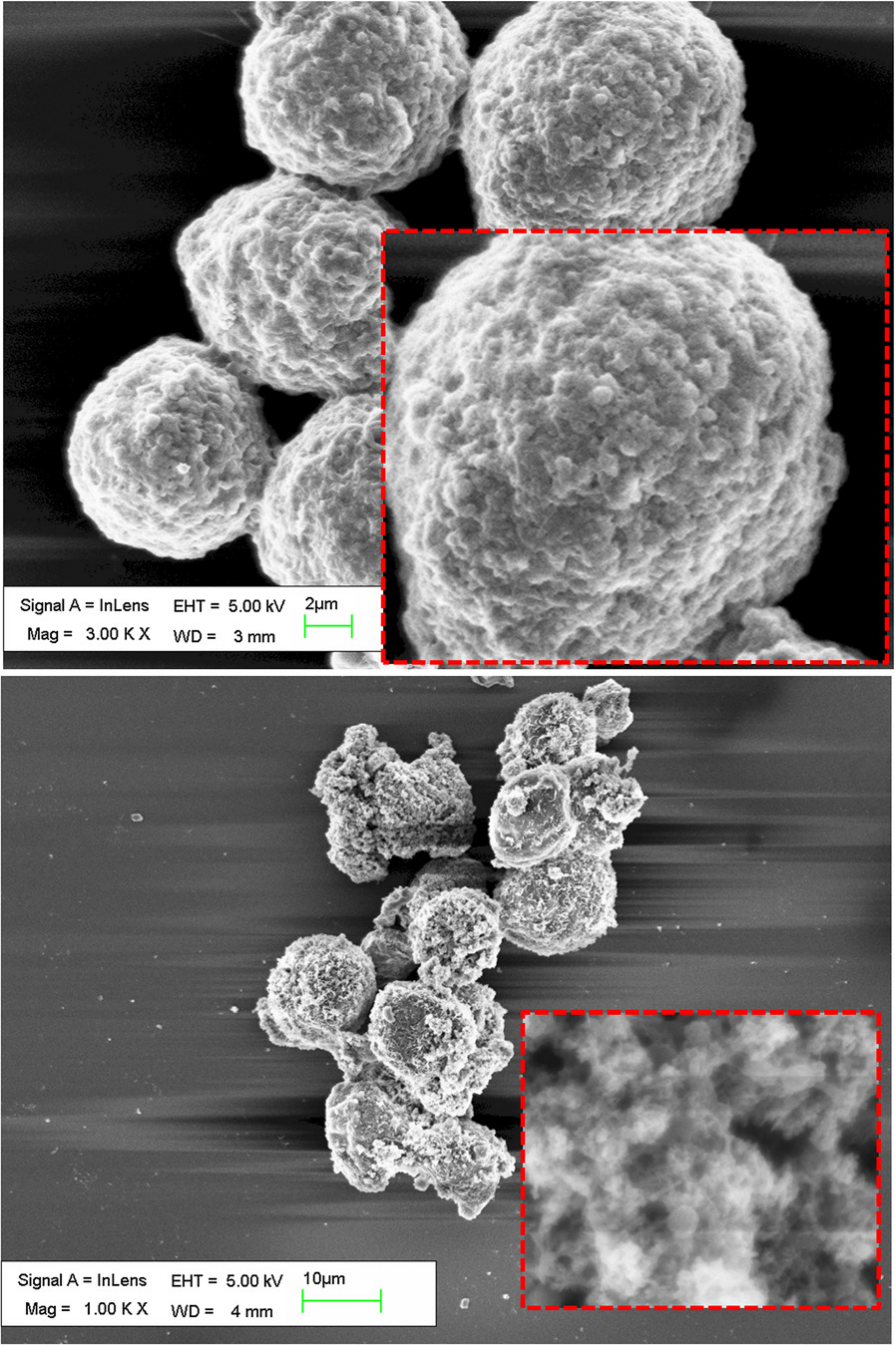
SEM images of rMNP-loaded cells membrane before (upper) and after (lower) 2 h AMF treatment.

**Figure 5:**
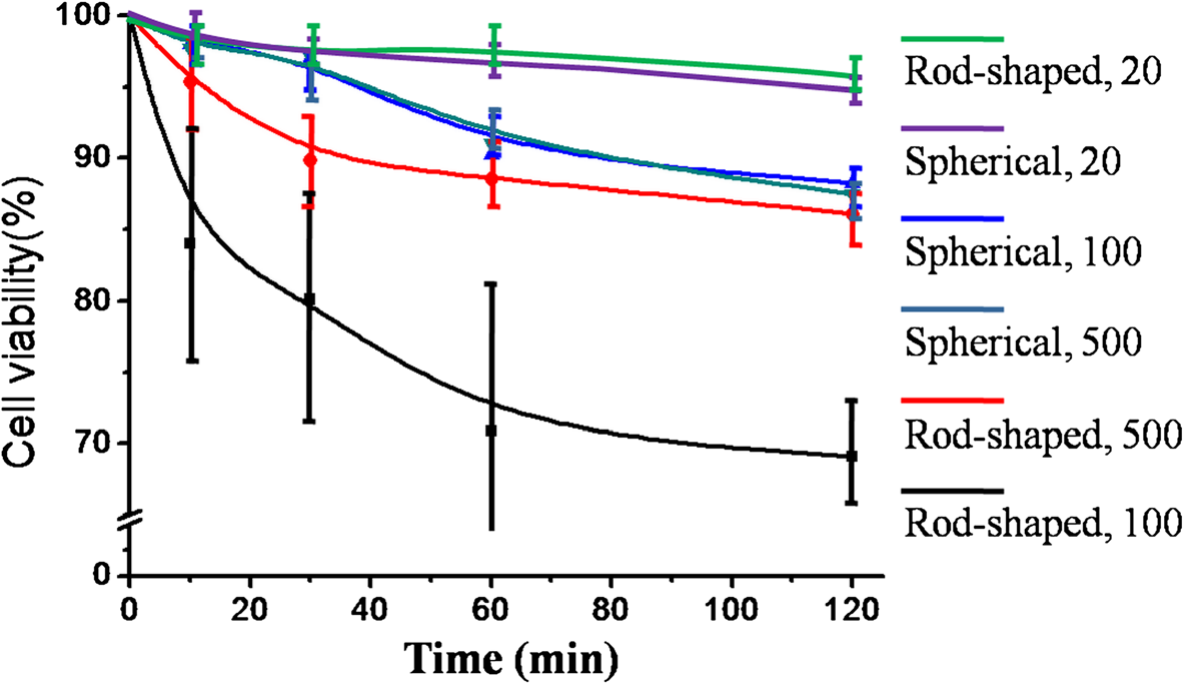
**Cell viability of MNP-loaded HeLa cells after AMF treatment for a while.** The corresponding morphologies and MNPs concentration in microgram per milliliter are listed on the right.

In this study, AMF treatment was approved of an obvious inactivation effect on MNP-loaded HeLa cells. As shown in Figure 4, forced vibration of MNPs mechanically destroys the cell membrane structure, leading to apoptosis. After AMF treatment, the relative viabilities of the MNP-loaded cells generally decreased. The effect of mechanical damage was not fully shown at the beginning period of AMF treatment. However, the efficiency increased because of the cumulative effect of mechanical oscillations. Hence, longer AMF treatment period is required in practice. Meantime, the amount of MNP loading heavily influenced the inactivation effect as well. Take the 2-h treatment as an example, at the concentration of 20 μg/mL, the decreasing rates of sMNP-loaded cells and rMNP-loaded cells were 3.7% and 1.5%. No significant effect of oscillating MNPs in killing cancerous cells was observed. At the concentration of 100 μg/mL, the corresponding decreasing rates were 11.7% and 30.9%, proving the morphological effect of MNPs. While at concentration of 500 μg/mL, 12.5% and 13.9% HeLa cells were killed by spherical MNPs and rod-shaped MNPs, respectively, but no significant difference was observed as well. The details of cell viability relative to AMF treatment time were shown in Figure 5. For the three concentrations of MNPs in this study, only the medium concentration was demonstrative of the morphological effect. For the interesting phenomenon that medium concentration was more suitable than higher or lower ones, we assume that it could be explained by the following two aspects. Firstly, the power of the device used in this study was too low to drive MNPs in high concentrations to oscillate inside cells or tissue efficiently and simultaneously, and too many particles in AMF had mutual restraint effect if they assembled in clusters, especially for rod-shaped MNPs. On the contrary, with low intake of MNPs, it was hard to effectively influence cell viability by mechanical oscillations.

**Figure 6:**
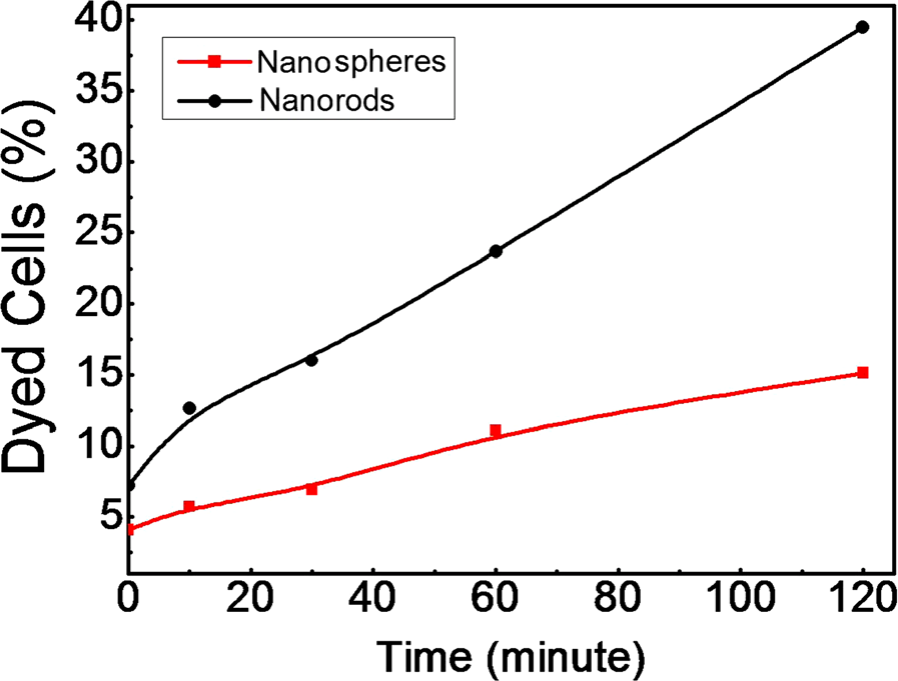
**Percentage of trypan blue-stained cells.** These cells had been pre-cultured in 100 μg/mL MNPs suspended culture medium and exposed to an AMF for up to 2 h.

It is supposed that MNPs embedded into the cell membranes mainly contributed to cell death by destroying the membranes. Cell dyeing is indicative of cell membrane damage. In this study, trypan blue assay, which was sensitive to permeability of membranes, was further used to verify the observed morphological effect at the concentration of 100 μg/mL. As shown in Figure 4, the HeLa cells that were incubated with 100 μg/mL rod-shaped MNPs appeared to have a loose cell structure after 2 h AMF treatment. For the 2-h groups, 39.47% of the rMNP-loaded cells were stained, while only 15.13% of sMNP-loaded cells were stained. Details of trypan blue staining were shown in Figure 6. This result is consistent with the observed decreases in cell viability. In a previous research, the concentration- and time-dependent damage of iron oxide MNPs to cell membrane injury was observed as well [23], supporting the concentration dependence of this study. The morphological effect was fully shown in this situation: rod-shaped MNPs pre-incubated with 100 μg/mL and placed in AMF for 2 h or more.

### Mechanisms of morphological effect

**Figure 7:**
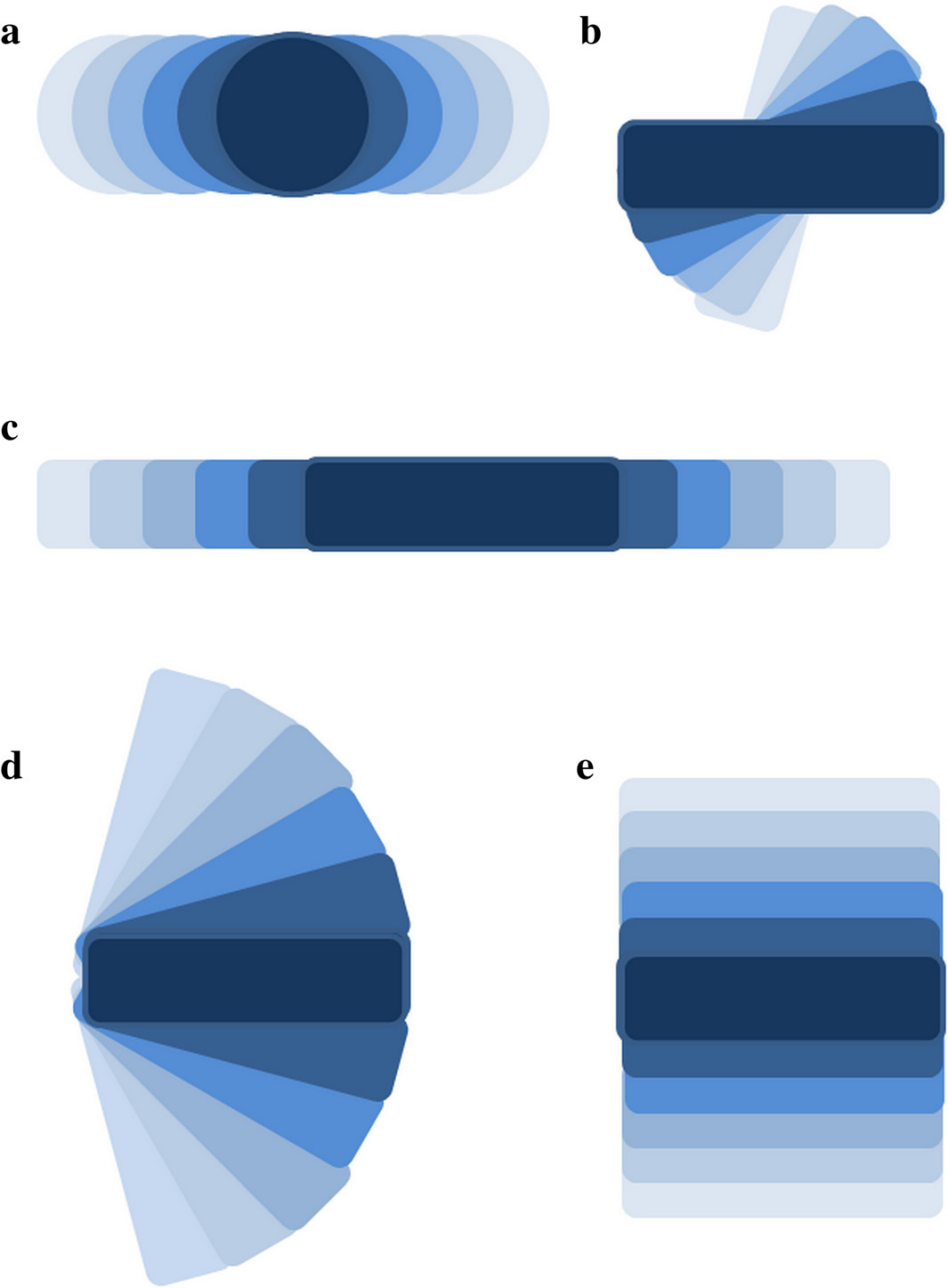
**Possible patterns of MNPs' forced oscillations.** There are more potential patterns of rMNPs than presented **(b, c, d, e)**, and the rMNPs' oscillations are often of a larger scope.

The results showed that MNP morphology and concentration have an important influence on the cell inactivation effects of AMF-assisted forced vibration of MNPs. The differences in cell inactivation efficiency may result from the difference of driving force moment. Relatively asymmetric morphology, such as rod-shaped, leads to greater magnetic torque, more intense oscillation and a larger involved area in AMF as shown in Figure 7. The morphological effect was indirectly reflected by the coercivity of the MNPs as well, which is related to the demagnetization effect. Though the saturation magnetic inductions were similar, the coercivity of the rod-shaped MNPs was 110.42 Gs, which is twice as much as the coercivity of the spherical MNPs (53.185 Gs). This suggests that the vibrations of rod-shaped MNPs consume more energy, i.e., more energy is used for mechanical movement when compared with the spherical MNPs. Additionally, the difference between sMNP and rMNP intakes (85% vs 89%) by HeLa cells may contribute to the morphological effects as well.

## Conclusions

In this research, AMF-induced oscillation of MNPs was proved able to mechanically damage cancer cells *in vitro*, especially when relatively asymmetric rod-shaped MNPs were used. Additionally, the concentration of MNPs affects the efficiency of AMF treatment. In this study, AMF treatment was most efficient when cells were in advance culture in medium containing MNPs at a concentration of 100 μg/mL and treated for 2 h or more.

## Competing interests

The authors declare that they have no competing interests.

## Authors' contributions

DC, XL, and GZ designed the experimental scheme and implement it; XL drafted the manuscript; GZ and HS modified the manuscript. All authors read and proved the final manuscript.

## References

[B1] Ahmed N Jaafar-Maalej C Eissa MM Fessi H Elaissari A New oil-in-water magnetic emulsion as contrast agent for in vivo magnetic resonance imaging (MRI) J Biomed Nanotechnol 2013 9 1579 1585 10.1166/jbn.2013.1644 23980505

[B2] Ge Y Zhang Y He S Nie F Teng G Gu N Fluorescence modified chitosan-coated magnetic nanoparticles for high-efficient cellular imaging Nanoscale Res Lett 2009 9 287 295 10.1007/s11671-008-9239-9 20596545 PMC2893437

[B3] Akbarzadeh A Samiei M Davaran S Magnetic nanoparticles: preparation, physical properties, and applications in biomedicine Nanoscale Res Lett 2012 9 144 156 10.1186/1556-276X-7-144 22348683 PMC3312841

[B4] Wahajuddin Arora S Superparamagnetic iron oxide nanoparticles: magnetic nanoplatforms as drug carriers Int J Nanomedicine 2012 9 3445 3471 22848170 10.2147/IJN.S30320 PMC3405876

[B5] Wang C Xu R Tang L The local heating effect by magnetic nanoparticles aggregate on support lipid bilayers J Biomed Nanotechnol 2013 9 1210 1215 10.1166/jbn.2013.1655 23909135

[B6] Samanta B Yan H Fischer NO Shi J Jerry DJ Rotello VM Protein-passivated Fe_3_O_4_ nanoparticles: low toxicity and rapid heating for thermal therapy J Mater Chem 2008 9 1204 1208 10.1039/b718745a 19122852 PMC2593465

[B7] Fortin JP Wilhelm C Servais J Ménager C Bacri JC Gazeau F Size-sorted anionic iron oxide nanomagnets as colloidal mediators for magnetic hyperthermia J Am Chem Soc 2007 9 2628 2635 10.1021/ja067457e 17266310

[B8] Silva AC Oliveira TR Mamani JB Malheiros SMF Malavolta L Pavon LF Sibov TT Amaro E Tannus A Vidoto ELG Martins MJ Santos RS Gamarra LF **Application of hyperthermia induced by superparamagnetic iron oxide nanoparticles in glioma treatment** Int J Nanomedicine 2011 9 591 603 21674016 10.2147/IJN.S14737 PMC3107718

[B9] Gonzalez-Fernandez MA Torres T Andrés-Vergés M Costo R Presa P Serna CJ Morales MP Marquina C Ibarra MR Goya GF Magnetic nanoparticles for power absorption: optimizing size, shape and magnetic properties J Solid State Chem 2009 9 2779 2784 10.1016/j.jssc.2009.07.047

[B10] Kim DH Rozhkova EA Ulasov IV Bader SD Rajh T Lesniak MS Novosad V Biofunctionalized magnetic-vortex microdiscs for targeted cancer-cell destruction Nat Mater 2010 9 165 171 10.1038/nmat2591 19946279 PMC2810356

[B11] Goya GF Fernandez-Pacheco R Arruebo M Cassinelli N Ibarra MR Brownian rotational relaxation and power absorption in magnetite nanoparticles J Magn Magn Mater 2007 9 132 135 10.1016/j.jmmm.2007.02.033

[B12] Dutz S Kettering M Hilger I Müller R Zeisberger M Magnetic multicore nanoparticles for hyperthermia–influence of particle immobilization in tumour tissue on magnetic properties Nanotechnology 2011 9 265102 10.1088/0957-4484/22/26/265102 21576784

[B13] Cho HS Dong Z Pauletti GM Zhang J Xu H Gu H Wang L Ewing RC Huth C Wang F Shi D Fluorescent, superparamagnetic nanospheres for drug storage, targeting, and imaging: a multifunctional nanocarrier system for cancer diagnosis and treatment ACS Nano 2010 9 5398 5404 10.1021/nn101000e 20707381

[B14] Dos Santos T Varela J Lynch I Salvati A Dawson KA Quantitative assessment of the comparative nanoparticle-uptake efficiency of a range of cell lines Small 2011 9 3341 3349 10.1002/smll.201101076 22009913

[B15] Chithrani BD Ghazani AA Chan WCW Determining the size and shape dependence of gold nanoparticle uptake into mammalian cells Nano Lett 2006 9 662 668 10.1021/nl052396o 16608261

[B16] Lu F Wu S-H Hung Y Mou CY Size effect on cell uptake in well-suspended, uniform mesoporous silica nanoparticles Small 2009 9 1408 1413 10.1002/smll.200900005 19296554

[B17] Lan X Cao X Qian W Gao W Zhao C Guo Y Long Fe_3_O_4_ nanowires decorated by CdTe quantum dots: synthesis and magnetic–optical properties J Solid State Chem 2007 9 2340 2345 10.1016/j.jssc.2007.06.007

[B18] Harima H Kawamura H Kitaoka Y Kohno H Miyake K Suzuki Y Sakakima H Zheng G Atsumi T Jeyadevan B Sato Y Tohji K Heating efficiency of magnetite particles exposed to AC magnetic field J Magn Magn Mater 2007 9 2841 2843 10.1016/j.jmmm.2006.11.063

[B19] Jia D Liu J Current devices for high-performance whole-body hyperthermia therapy Expert Rev Med Devices 2010 9 407 423 10.1586/erd.10.13 20420562

[B20] Mukherjee P Cherukuri P Glazer ES Curley SA Targeted hyperthermia using metal nanoparticles Adv Drug Deliv Rev 2010 9 339 345 10.1016/j.addr.2009.11.006 19909777 PMC2827640

[B21] Hirsch LR Stafford RJ Bankson JA Sershen SR Rivera B Price RE Hazle JD Halas NJ West JL Nanoshell-mediated near-infrared thermal therapy of tumors under magnetic resonance guidance Proc Natl Acad Sci U S A 2003 9 13549 13554 10.1073/pnas.2232479100 14597719 PMC263851

[B22] Häfeli U Zborowski M Tomitaka A Hirukawa A Yamada T Morishita S Takemura Y Biocompatibility of various ferrite nanoparticles evaluated by in vitro cytotoxicity assays using HeLa cells J Magn Magn Mater 2009 9 1482 1484 10.1016/j.jmmm.2009.02.058

[B23] Naqvi S Samim M Abdin M Ahmed FJ Maitra A Prashant C Dinda AK Concentration-dependent toxicity of iron oxide nanoparticles mediated by increased oxidative stress Int J Nanomedicine 2010 9 983 989 21187917 10.2147/IJN.S13244 PMC3010160

